# Thermoelectric Response Enhanced by Surface/Edge States in Physical Nanogaps

**DOI:** 10.3390/ma16020660

**Published:** 2023-01-10

**Authors:** Víctor Manuel García-Suárez

**Affiliations:** Department of Physics, University of Oviedo & CINN, 33007 Oviedo, Spain; vm.garcia@cinn.es

**Keywords:** thermoelectricity, nanogaps, surfaces, 2D materials, tight-binding

## Abstract

Current solid-state thermoelectric converters have poor performance, which typically renders them useless for practical applications. This problem is evidenced by the small figures of merit of typical thermoelectric materials, which tend to be much smaller than 1. Increasing this parameter is then key for the development of functional devices in technologically viable applications that can work optimally. We propose here a feasible and effective design of new thermoelectric systems based on physical gaps in nanoscale junctions. We show that, depending on the type of features, i.e., the character of surface/edge states, on both sides of the gap, it is possible to achieve high figures of merit. In particular, we show that, for configurations that have localized states at the surfaces/edges, which translate into sharp resonances in the transmission, it is possible to achieve large Seebeck coefficients and figures of merit by carefully tuning their energy and their coupling to other states. We calculate the thermoelectric coefficients as a function of different parameters and find non-obvious behaviors, such as the existence of a certain coupling between the localized and bulk states for which these quantities have a maximum. The highest Seebeck coefficients and figures of merit are achieved for symmetric junctions, which have the same coupling between the localized state and the bulk states on both sides of the gap. The features and trends of the thermoelectric properties and their changes with various parameters that we find here can be applied not only to systems with nanogaps but also to many other nanoscale junctions, such as those that have surface states or states localized near the contacts between the nanoscale object and the electrodes. The model presented here can, therefore, be used to characterize and predict the thermoelectric properties of many different nanoscale junctions and can also serve as a guide for studying other systems. These results pave the way for the design and fabrication of stable next-generation thermoelectric devices with robust features and improved performance.

## 1. Introduction

Solid state thermoelectric converters are key to designing systems that involve both the conversion of electricity to heat in charge-driven cooling systems and of heat to electricity in heat-driven current generators. These systems are essential for different industrial and technological processes, which range from applications in the automotive industry to space applications, biotechnological applications, and wastes of heat in industrial processes [1,2,3,4,5,6,7,8]. Improving current thermoelectric converters and designing new ones with enhanced capabilities is expected to have a substantial impact in several scientific and technological areas and to help to achieve different objectives related to energy saving and transformation, green energies and zero-carbon emissions [4].

Current solid state thermoelectric converters can already be used in specialized applications, such as spacecraft [9]; however, many of them present a series of problems that reduce their performance and make them impractical for certain technological applications. Among these problems, the small efficiency and poor performance of many of such systems integrated in different devices stand out.

For instance, the largest figures of merit in present-day solid state converters are of the order of 1 [10,11,12,13,14], with the highest ones achieved up to now of around 3 [15,16,17]. These values can be reached mainly for bulk materials with nanostructured lattices. However, such values are clearly not sufficient to achieve efficient thermoelectric conversion nor fabricate efficient and effective converters to be employed in applications for real-life systems.

The main issues that severely affect the thermoelectric performance and lead to very poor performances come, on one hand, from the relatively small Seebeck coefficients, and, on the other hand, from the relatively large phonon conductances of most solid-state thermoelectric materials. The first factor is key to transform a temperature gradient into a potential difference by means of the Seebeck effect (or the other way round, through the Peltier effect), and the second factor, which is added to the electronic thermal conductance [18], is key to ensure, when it is small enough, that the temperature gradient is kept and does not vanish, so that the thermal induced voltage difference or electrical current is maintained. However, the relatively small Seebeck coefficients and large phonon conductances of most solid state converters render them useless or unable to work properly, with poor performance compared to other devices used in the recent past.

In order to solve the problems that severely affect current devices and hamper their efficiency, various approaches that tackle different physical aspects have been proposed and implemented over the past years. These approaches include, for instance, the increase of the scattering of phonons in materials that include different sublattices or the nanostructuration of materials in different ways [19,20]. This last approach in particular, i.e., nanostructuring certain parts or a whole material by including nanometric defects, has proven to be promising for designing new materials with high Seebeck coefficients (*S*) and figures of merit (ZT).

Some examples include the use of nanoscale junctions, such as molecular electronic systems with high ZT [21], or two dimensional materials, such as graphene (see, e.g., [22,23,24] and the references therein). However, such systems are difficult to fabricate in practice, and their relatively high figures of merit can only be reached within a very narrow and unstable window of parameters. In addition, in most of the calculations used to model such materials and predict their thermoelectric properties, the phonon thermal conductance, which is an important limiting factor that can severely reduce the size of ZT, is not considered and, therefore, it is not clear in many cases how efficiently these systems can work as thermoelectric converters.

In this work, we propose the development of efficient nanoscale thermoelectric systems based on physical gaps between surfaces of bulk materials or 2D layers. We stress that such systems are real and some of them, such as those based on graphene layers, have already been characterized experimentally to an extent and simulated with ab initio techniques [25]. The proposed model then describes a specific type of physical systems, and we study the main parameters that affect the thermoelectric performance.

We describe and model the properties of these systems and show under which conditions they display the highest performance. Such systems include, as commented before, graphene gaps, which can be fabricated with electroburning techniques [26,27,28,29] or with mechanically controllable break junctions [30] and are currently being profusely studied by several groups worldwide as possible platforms to design new molecular electronics and nanoscale components. Examples of these last devices comprise the coupling of molecular wires, crossover molecules or other similar systems between graphene electrodes.

An interesting property of such nanoscale devices is that, even without any bridging element, they are expected to be reliable and display, in certain cases, a good thermoelectric response generated, on one hand by the sharp transmission produced by different features on the edges of both sides of the gap [31], as we shall see, and, on the other hand, by the lack of phonon transport across physically separated electrodes. The first factor, which is typical for instances of systems, such as heavily doped semiconductors [32], is key to generating large Seebeck coefficients, while the second one leads to the elimination of an important limiting effect in most solid-state thermoelectric materials. We note, however, that, in some cases, phonons can still cross the gap and give sizeable thermal conductances [33]. This has also been studied recently in different systems, and it has been found in many cases that such conductances cannot be ignored [34,35,36,37,38,39]. We will, therefore, use an approximation to calculate such contributions (see below).

We show then that, in stable designs and under certain conditions, it is possible to achieve very high *S* and ZT, which should allow building nanoscale devices with high efficiencies. Such designs could also be able to be achieved in the near future due to the rapid advance of the fabrication and characterization techniques of nanogaps [26,27,28,29] in two-dimensional materials and related systems. The delivery of edges with tailored shapes and morphologies should also allow the implementation of several of the proposed systems in parallel, which would then multiply the thermoelectric performance and help to scale and integrate these systems into technologically relevant devices.

The article is structured as follows: In Section 2, we describe the tight-binding model that we use to describe different configurations and calculate their thermoelectric properties. In Section 3, we present the results for two different setups: a wedge/adatom or protuberance on one side and a straight edge/flat surface on the other and wedges/adatoms or protuberances on both sides. We finish with our summary and conclusions.

## 2. Materials and Methods

**Figure 1:**
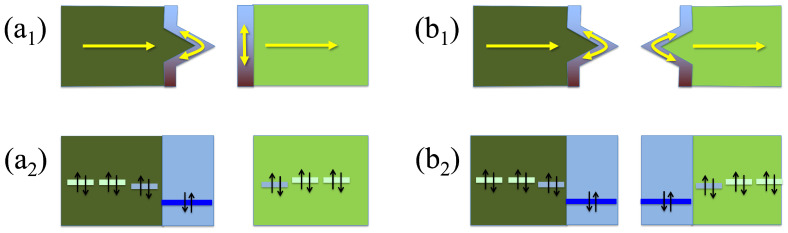
Schematic representation of the wedge–edge (**a**) and wedge–wedge (**b**) configurations. The top panels (1) show a physical representation of electrons flowing through 2D layers due to a bias voltage or a temperature gradient and the electrons on the edge states, which do not couple to the former when their group velocity is perpendicular; the bottom panels (2) show the corresponding levels of the tight-binding model.

We describe the system, which is comprised of either two facing surfaces of a bulk material or two facing edges of a 2D material, both of them separated by a gap and with or without wedges or protuberances, with a tight-binding model that considers the coupling between the bulk states and those states belonging to wedges/adatoms or protuberances as well as the coupling between those states across the gap [25,31]. An schematic representation of the system and the theoretical model are shown in Figure 1. The model is comprised of two one-dimensional semi-infinite chains that end in a surface state and couple across a gap. We notice and stress that, even though this may seem to be an oversimplified system, it perfectly captures the transport properties of these or similar systems, and gives results that agree almost exactly with those calculated using more involved ab initio simulations [25,31].

The model works properly because it considers the essential parameters that affect the electronic transport through the system. As such, it is able to accurately reproduce the transport characteristics around the Fermi level at zero bias and also at relatively large bias voltages, giving good agreements for all those regimes with the aforementioned ab initio simulations and even with experiments [25]. We also note that the model is dynamically stable since it does not evolve over time, i.e., the sites and all parameters are kept fixed and do not change; this is relevant for systems with strong bonds whose coordinates do not change at room temperature. Therefore, it is not necessary to perform tight-binding molecular dynamics [40].

The Hamiltonian includes two electrodes that couple through a vacuum gap: $\hat{H}={\hat{H}}_{l}+{\hat{H}}_{r}+{\hat{V}}_{\mathrm{lr}}$, where l and r stand for left and right electrodes, respectively. This Hamiltonian includes only electronic terms—but not phonons—because, as commented before, the transport of phonons should be negligible due to the vacuum gap between the electrodes. The most important parameters in this model are the strength of the couplings between the edge/surface states and the bulk states and the strength of the coupling between bulk or edge/surface states across the gap. The Hamiltonian of both sides (left and right), ${\hat{H}}_{l(r)}$, is defined as follows:

(1) $$\begin{array}{cc} {\hat{H}}_{l(r)} & =\sum_{\langle i,j\rangle ;\sigma }t_{ij\sigma }{\hat{c}}_{l(r),i\sigma }^{†}{\hat{c}}_{l(r),j\sigma }+\sum_{\sigma }\epsilon_{\sigma }{\hat{d}}_{l(r),\sigma }^{†}{\hat{d}}_{l(r),\sigma }+\\ \; & +\sum_{\sigma }t_{1d\sigma }\left({\hat{c}}_{l(r),1\sigma }^{†}{\hat{d}}_{l(r),\sigma }+h.c.\right) \end{array}$$

where ${\hat{c}}_{l(r),i\sigma }$ and ${\hat{d}}_{l(r),\sigma }$ represent the annihilation operators on the bulk and edge/surface states on the left (right) electrode for spin σ (σ = ↑, ↓), respectively, $t_{ij\sigma }$ are the spin-dependent on-site energies ($t_{ii\sigma }=\epsilon_{\sigma }$) and coupling terms between different sites and the sum $\langle i,j\rangle$ runs only to first nearest neighbors. Notice that we assume the electronic repulsion at the surface state is sufficiently small to avoid including a large intra-atomic repulsion *U*, which means the model might not apply to strongly correlated systems with localized states and large *U* (i.e., d or f states), where it would be necessary to go beyond a single particle description. This is generally true for surface states where electrons are delocalized between various atoms.

In this case, we take a mean field approach, and *U* is included as a shift of the on-site energy level. The states at the surface or edges can have a magnetic configuration, i.e., a spin-splitting $|\epsilon_{↑}\left|\neq \right|\epsilon_{↓}|$. In such a case, the bulk states closer to them can also be affected, which may then translate into an on-site energy slightly different from that of the other bulk states and even a small magnetic splitting ($t_{11↑}\neq t_{11↓}\neq \epsilon_{\sigma }$). We use $t_{ij\sigma }=t=-3$ eV and $\epsilon_{\sigma }=\epsilon =0$ eV (the same values for both spins). To reflect the influence of the surface on the states close to it, we apply a shift to $t_{11}=\epsilon_{1}$ of −0.05 eV. We use, for the surface state, an on-site energy level ϵ=−0.5 eV, which defines the position of the resonance in the transmission.

The other term that enters in the Hamiltonian couples on both sides of the gap and is given by

(2) $$\begin{array}{cc} {\hat{V}}_{\mathrm{lr}} & =\sum_{\sigma }[\gamma_{\mathrm{dd}\sigma }{\hat{d}}_{l,\sigma }^{†}{\hat{d}}_{r,\sigma }+\sum_{i,j=1}^{2}(\gamma_{di\sigma }{\hat{d}}_{l,\sigma }^{†}{\hat{c}}_{r,i\sigma }+\gamma_{id\sigma }{\hat{c}}_{l,i\sigma }^{†}{\hat{d}}_{r,\sigma }+\\ \; & +\gamma_{ij\sigma }{\hat{c}}_{l,i\sigma }^{†}{\hat{c}}_{r,j\sigma })+h.c.]. \end{array}$$

This part considers the couplings between bulk and/or localized states at the edge/surface across the gap. The first (second) index of the coupling elements γ (*d* or *i*) corresponds to a localized or bulk state in the left (right) part and vice versa.

This last term of the Hamiltonian and the previous couplings between the bulk and surface states give rise, when computed the transmission, to features that lead to remarkable electronic and thermoelectric characteristics, as we will see later. Notice that the total Hamiltonian has also been slightly simplified with respect to the one used in previous studies, which was employed to calculate the electronic transport properties with more configurations and combinations of parameters. In this case, however, we keep only the most relevant terms that affect the thermoelectric performance.

Other parameters, such as second order couplings or other couplings to the surface states that generate interference effects and that can appear in particular cases [31], are not considered in this Hamiltonian, because they mainly influence the transmission and the current but have not much effect on certain thermoelectric properties (the Seebeck coefficient and figure of merit). We keep the coupling between the closest states across the gap, which can be, depending on the configuration, a surface and a bulk state or two bulk states; we use the same value of the coupling in both cases, i.e., $\gamma_{d1}=\gamma_{\mathrm{dd}}=-0.01$ eV, respectively.

The thermoelectric coefficients are obtained by first calculating the energy-dependent transmission T(E) through the junction,

(3) $$T\left(E\right)=\mathrm{Tr}\left[\Gamma_{L}\;G_{M}^{R†}\;\Gamma_{R}\;G_{M}^{R}\right]\left(E\right)$$

where $G_{M}^{R}\left(E\right)={\left[E+i\delta -H-\Sigma_{L}^{R}\left(E\right)-\Sigma_{R}^{R}\left(E\right)\right]}^{-1}$ is the retarded Green’s function of the scattering region, which includes part of the electrodes, $\Gamma_{\alpha }=i\left[\Sigma_{\alpha }^{R}-\Sigma_{\alpha }^{R†}\right]$ and $\Sigma_{\alpha }^{R}\left(E\right)=V_{M\alpha }\;G_{\alpha }^{0R}\;V_{M\alpha }=t_{ij\sigma }^{2}\;G_{\alpha }^{0R}$ is the corresponding self energy for electrode α, with $G_{\alpha }^{0R}$ the bulk retarded Green’s function of the electrodes. For this particular case, $G^{0R}=2/\left(E-\epsilon +\Delta \right)$, where $\Delta =\mathrm{sign}\left(E-\epsilon \right)\sqrt{\left(E-\epsilon^{2}\right)-4\;t^{2}}$—the same for both electrodes. After calculating the transmission, the next step involves the evaluation of the moments of the transmission using well-known expressions that consider the transmission and the Fermi distribution function [21], i.e., by extending the Landauer–Büttiker formalism to consider both charge and heat currents [41].

The transport is ballistic, and therefore the expressions for the thermoelectric coefficients do not depend on parameters such as the electron relaxation time. The temperature dependence enters into the Fermi distribution function or, more explicitly, into its derivative within the expressions of the moments of the transmission ($L_{n}\left(T\right)=\int_{-\infty }^{\infty }{\left(E-E_{F}\right)}^{n}T\left(E\right)\left[\partial f\left(E,T\right)/\partial E\right]dE$, where $E_{F}$ is the Fermi energy and *f* is the Fermi distribution function). The conductance, Seebeck coefficient, electronic thermal conductance and figure of merit are given in terms of moments, such as

(4) $$G=\frac{2e^{2}}{h}L_{0}$$

(5) $$S=-\frac{1}{eT}\frac{L_{1}}{L_{0}}$$

(6) $$\kappa =\frac{2}{hT}\left(L_{2}-\frac{L_{1}^{2}}{L_{0}}\right)$$

(7) $$ZT=\frac{S^{2}GT}{\kappa }=\frac{1}{\frac{L_{0}L_{2}}{L_{1}^{2}}-1}$$

and are then evaluated at particular values of the Fermi energy and temperature. In the figure of merit, we also consider the effect of the phonon conductance, which enters as an additional factor in the denominator:

(8) $$ZT=\frac{S^{2}GT}{\kappa +\kappa_{\mathrm{ph}}}$$

We approximate such a term by using a conductance per unit area of $10^{7}$ W/(m${}^{2}$K), which is a typical value that can be found for distances around 3 Å at room temperature [39], and an area of 0.25 nm${}^{2}$ (an square with a side of 0.5 nm, which is still relatively large for systems that have a few atoms at the tip). This gives a thermal conductance $\kappa_{\mathrm{ph}}=2.5$ pW/K.

The model considers three possible scenarios that cover the most relevant configurations of physical nanogaps between surfaces or edges of 2D materials. These configurations, which generate a plethora of electronic effects even without any bridging component, depend on the particular structure of the edges/surfaces on both sides of the junction and produce completely different transport properties. The main difference between such configurations is due to the absence or presence of structural defects, such as protuberances or wedges, on one or both sides. These structural differences translate in the Hamiltonian in changes of the coupling between the bulk and the surface states ($t_{l(r),1d\sigma }$); this coupling is zero for straight edges/flat surfaces or has a particular finite value for wedges/adatoms or protuberances.

When such a coupling is not zero, it can give rise to sharp transmission resonances that signal the presence of localized states at the edges. The slope of such resonances can dramatically modify the Seebeck coefficient and, consequently, the figure of merit, following, thus, the strategy proposed by Mahan and Sofo to achieve the best thermoelectric performance [42]. We will focus then on cases that give rise to such resonances, i.e., those with wedges/adatoms on one one or both sides of the gap.

## 3. Results and Discussion

We calculated results for all possible configurations that stem from different combinations of features on both sides of the gap. As commented before, these features can be wedges that protrude from the edges of two-dimensional materials or adatoms/protuberances that protrude from the surfaces of three-dimensional materials. The possible combination of these features with straight edges or surfaces gives three possible cases [25,31]. However, the simplest case, which has straight edges or clean surfaces on both sides of the gap and leads to smooth and featureless transmissions, produces rather small magnitudes of the thermoelectric coefficients and poor efficiencies as demonstrated by the small figure of merit, and thus we will not consider it.

We will focus then on the other two cases, which have sharp features that can potentially lead to much higher thermoelectric coefficients: the wedge–edge (we use this notation, which refers two-dimensional materials; however, the model also applies, as commented previously, to surfaces of three-dimensional materials with or without protrusions or adatoms [31]) and wedge–wedge configurations. Notice also that the wedge–edge configuration has the largest probability of occurring in the current experimental setups and has even been measured in recent experiments [25]. We model, as commented before, non-magnetic configurations, which give a single peak in the transmission; we use, in this case, a surface on-site energy level ϵ=−0.5 eV and an additional shift of the states close to this of −0.05 eV (see above).

Finally, in addition to the purely theoretical results, and in order to compare with these, we have also included in Appendix A ab initio results of the thermoelectric properties of nanogaps between graphene layers. Notice that in this case the electronic states are spin-split because the graphene edges have magnetic configurations.

### 3.1. Wedge–Edge Configuration

The zero-bias transport properties in this case are characterized by the presence of one or two Breit–Wigner resonances in the transmission, depending on whether the system has spin polarization. Such resonances can be located at a given energy relative to the Fermi level and have a certain width, depending on a series of parameters that enter in the model. The position is determined by the on-site energies of the surface/edge states, and the width is governed by the coupling to the bulk states. The location and sharpness of these states are key to the generation of large Seebeck coefficients and figures of merit, since these quantities increase with the slope of the transmission at the Fermi level.

As a function of the Fermi energy, which we assume can be smoothly changed with a gate potential, the conductance roughly follows the transmission, i.e., it has a structure with a single peak—as with a Breit–Wigner resonance. Regarding the Fermi level, however, in this case and in the following, the shifts of the Fermi level are somewhat exaggerated to clearly show the shape of the coefficients. In reality, it is not possible to produce such large shifts (larger than ∼2 eV) even with graphene electrodes that can be relatively close to a gate. The Seebeck coefficient changes non-monotonically and has a minimum below the on-site level (−0.5 eV) and a maximum above it, both of the same size.

This particular structure comes from the derivative of the Transmission, i.e., from its slope. The thermal conductance has a structure that is similar to the electrical conductance; however, this can change with the couplings, as we will see later. These electrical conductances, Seebeck coefficients and thermal conductances translate into a figure of merit ZT, which has two equal peaks as a function of the Fermi energy. Such peaks come mainly from the Seebeck coefficient *S*, which is squared in the numerator of ZT and is the main driving factor behind the thermoelectric performance as we will see.

#### 3.1.1. Evolution with the Coupling Parameter

The most important parameter that determines the sharpness of the resonances (which is one of the two main ingredients that, along with the position relative to the Fermi level, enhances the thermoelectric performance) is the coupling between the surface state and the bulk states. This parameter, $t_{l(r),1d\sigma }$, which couples the localized state to the bulk states on each side, depends on the type of material and the structural configuration (size and shape of the wedge/protusion; for instance, smaller or wider wedges in graphene give rise to smaller couplings between the edge states and the bulk states) [31].

Although, in reality, it cannot be changed continuously, the study of its evolution can help to determine the type of material and features that would be needed to generate the highest thermoelectric performances. This coupling parameter increases, in general, as the feature on the surface/edge (wedge/protrusion) becomes sharper, since it enhances the coupling between the edge state and the bulk states. The increase of the coupling, however, translates into an increase of the resonance width and, therefore, into a decrease of the thermoelectric performance. However, the picture is not that simple, and some details have to be considered as we shall see.

**Figure 2:**
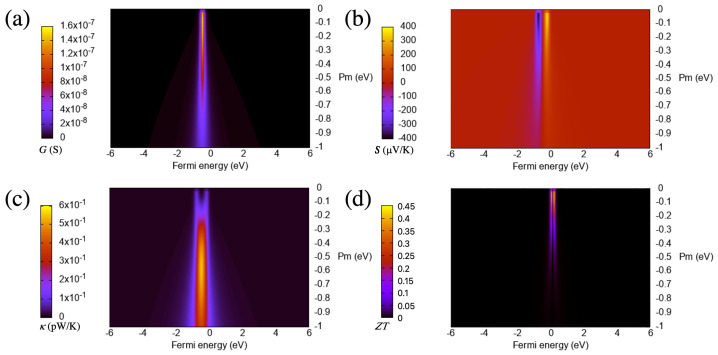
Electrical conductance (**a**), Seebeck coefficient (**b**), electronic thermal conductance (**c**) and figure of merit (**d**) as a function of the Fermi energy and the coupling parameter for the wedge–edge configuration. The temperature is 300 K.

**Figure 3:**
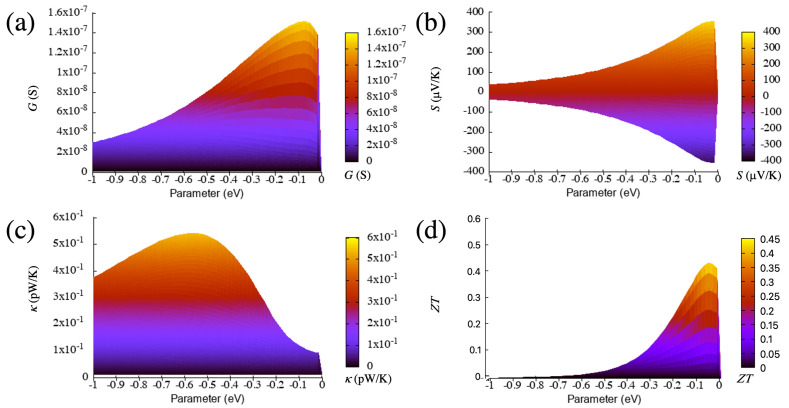
Electrical conductance (**a**), Seebeck coefficient (**b**), electronic thermal conductance (**c**) and figure of merit (**d**) as a function of the coupling parameter for the wedge–edge configuration (side view of Figure 2). The temperature is 300 K.

From the curves of Figure 2 and Figure 3, which are calculated at room temperature (300 K), it is clear that increasing the absolute value of the coupling parameter sharply increases the maximum of the conductance at the beginning but then steadily reduces it, with a slope that decreases for larger values. This means that such a maximum does not saturate nor remain constant but decreases as the coupling increases after an absolute value of around 0.1 eV. This behavior of the maximum of the zero-bias conductance with the coupling parameter can be reproduced by using an analytical model for the transmission of a system with one or two resonances on each side of the gap [43]:

(9) $$T\left(E\right)=\frac{4\Gamma_{L}\Gamma_{R}\gamma^{2}}{{\left(E_{\epsilon_{1}}E_{\epsilon_{2}}-\Gamma_{L}\Gamma_{R}-\gamma^{2}\right)}^{2}+{\left(E_{\epsilon_{1}}\Gamma_{R}+E_{\epsilon_{2}}\Gamma_{L}\right)}^{2}},$$

where $E_{\epsilon_{1(2)}}=E-\epsilon_{1(2)}$, $\epsilon_{1(2)}$ is the on-site energy of the level that couples to the left (right) electrodes, γ is the coupling of the levels across the gap and $\Gamma_{L(R)}=t_{L(R)}^{2}\rho_{L(R)}$, with $t_{L(R)}$ as the coupling between the levels and the corresponding lead and $\rho_{L(R)}$ as the density of states in each lead (we assume here a wide band gap approximation; therefore, this term is constant). In this case, the coupling to the right lead ($t_{R}$) is chosen to be smaller than that to the left lead, since, for this configuration, the right level is weakly coupled to the bulk states.

Notice as well that the conductance here is taken as the transmission at the Fermi level; in particular, when the Fermi energy coincides with the on-site energy level, i.e., by evaluating Equation (9) at the maximum ($E=\epsilon_{1}$) and also considering that, in these cases, $\epsilon_{1}=\epsilon_{2}$ gives $T\left(\epsilon_{1}\right)=4\Gamma_{L}\Gamma_{R}\gamma^{2}/{\left(-\Gamma_{L}\Gamma_{R}-\gamma^{2}\right)}^{2}$ and produces a curve that, as a function of the coupling parameter γ, correctly fits that shown in panel (a) of Figure 3 (and the corresponding one for the wedge-wedge case, see below). The maximum of such curve as a function of γ is located at $\gamma =-\sqrt{\Gamma_{L}\Gamma_{R}}$ and can therefore be changed by modifying the relative size of the couplings to the electrodes.

Note that the main difference between this and the next case (wedge–wedge) is the size of the coupling to the right electrode ($t_{R}$), which, as commented before, is smaller for this configuration. According to this model, the more asymmetric the couplings to the electrodes, the steeper the increase of the conductance curve at small γ, in agreement with what is shown in Figure 3 (compare this with the corresponding evolution for the wedge-wedge case, shown below). Such changes in the coupling give rise not only to different conductances but also to different thermoelectric properties (both quantitatively and qualitatively) and can therefore be considered key factors that substantially affect the thermoelectric performance. Note as well that these results are general can apply to many other nanoscale junctions that have similar resonances in the zero-bias transmission, such as those with surface states or with frontier orbitals near the contacts between the nanoscale element and the electrodes (for instance, molecular electronics systems or other nanoelectronic systems with different elements bridging the electrodes).

The increase of the coupling (in absolute terms) also enhances the magnitude of the Seebeck coefficient at the beginning as can be seen in Figure 3. However, for larger couplings, this quantity decreases due to the reduction of the slope of the transmission at the Fermi level (i.e., the resonance becomes wider and smoother when the coupling increases). This behavior can also be captured to a great extent by using, as a starting point, Formula (9) and calculating the expression for the Seebeck coefficient in terms of the transmission, which involves taking its derivative and dividing by the transmission [21]. This can also be easily verified in the zero-temperature limit by evaluating the expression at the Fermi energy, which gives a dependence on the derivative of the transmission (slope) divided by the transmission at that level.

At finite temperatures, however, the variation of the Seebeck coefficient with the coupling deviates slightly from this behavior, because the integration in energy that gives the momenta of the transmission decreases the value of the Seebeck coefficient for very small couplings, i.e., for very sharp resonances, and masks large Seebeck coefficients in such a limit. All evolutions with temperature are similar up to very large values, and the qualitative trend is maintained. In general, the smaller the temperature, the sharper the features (peaks and valleys) that develop in the thermoelectric coefficients—however, the smaller the values of the maxima and minima that such coefficients reach, as we will see in the next subsection.

The thermal conductance has two equally and clearly separated maxima for small (in absolute terms) couplings, which tend to merge as the magnitude of the coupling increases as can be seen in Figure 2. This behavior is not observed for the electrical conductance, whose shape usually follows that of the thermal conductance in many cases. The reason behind this tendency is the presence of the first moment of the transmission in the expression of the thermal conductance, which is a term that has, for small couplings, two peaks and therefore gives rise to a thermal conductance evolution with a similar pattern.

The figure of merit, which can also be seen in Figure 3, sharply increases first as a function of the absolute value of the coupling but then decreases steadily for larger values. The drop is a consequence of the increase of the thermal conductance first (from 0 to −0.5 eV), and the decrease of the electrical conductance and the Seebeck coefficient for values smaller than approximately −0.05 eV. This evolution gives rise to a maximum of ZT close to 0 eV. The values at or near the maximum are high (around 40), which lead to a huge thermoelectric performance. Such performance is then reached for a particular value of the coupling, which, in this case, is small (in absolute terms).

#### 3.1.2. Evolution with Temperature

Another important factor that influences the thermoelectric performance and can improve or worsen the response, is the temperature. The effect of this parameter, which enters into the Fermi distribution function, is known and has also been considered in past studies (see, e.g., [44]); however, its influence on the thermoelectric coefficients in the configurations considered in this study and the differences between them are not obvious and show interesting trends. We then also considered the evolution with temperature of all thermoelectric quantities.

**Figure 4:**
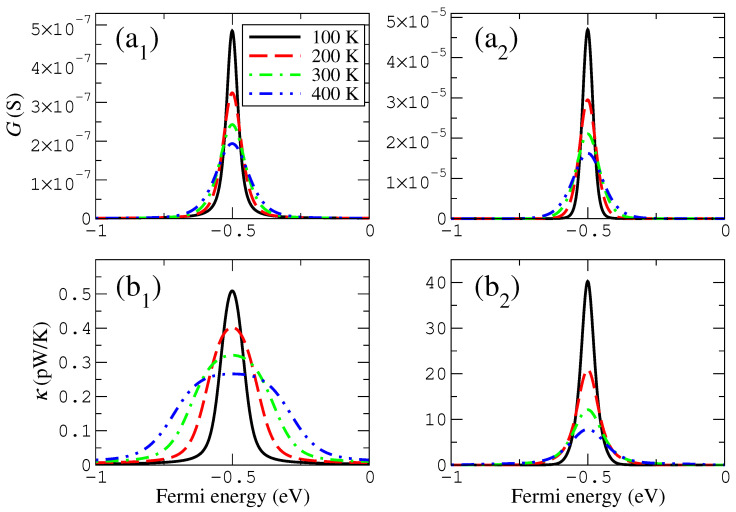
Temperature dependence of the electronic (**a**) and thermal (**b**) conductances for the wedge–edge (1) and wedge–wedge (2) configurations. The coupling parameter in this case is −0.2 eV. Notice that the vertical axis intervals in panels (1) and (2) are different.

**Figure 5:**
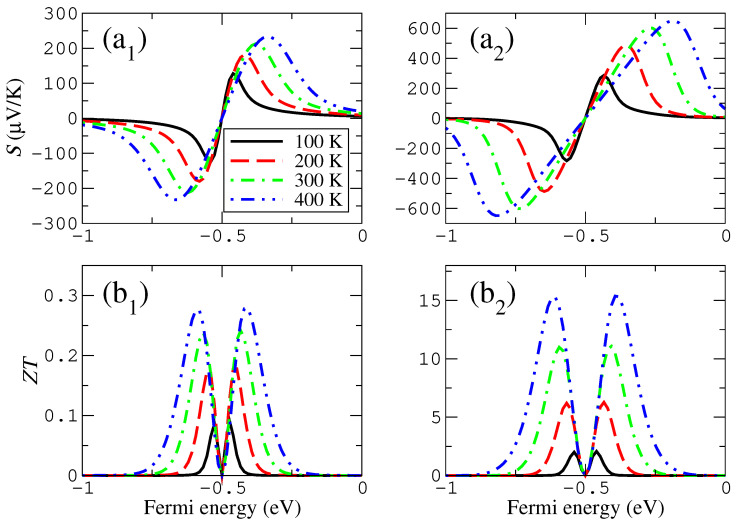
Temperature dependence of the Seebeck coefficient (**a**) and figure of merit (**b**) for the wedge–edge (1) and wedge–wedge (2) configurations. The coupling parameter in this case is −0.2 eV. Notice that the vertical axis intervals in panels (1) and (2) are different.

In particular, we calculated and show in Figure 4 and Figure 5 the change of the thermoelectric coefficients, grouping those that look similar (electronic and thermal conductances in Figure 4) and those that show more different trends (the Seebeck coefficient and the figure of merit in Figure 5) in a range of temperatures that can be accessed experimentally or can be meaningful for technological applications. Notice that these quantities are expected to clearly depend on temperature, since the transmission features around the Fermi level certainly feel the effect of the Fermi distribution function when the thermoelectric coefficients are calculated.

We found that the peaks of both the electrical and thermal conductances as a function of the Fermi energy widen and decrease as a function of temperature, although such trends are somewhat more pronounced in case of the electrical conductance. These evolutions can be easily understood, at least in case of the electrical conductance, by considering the widening of the resonance given by the derivative of the Fermi distribution function that enters in the calculation for such quantities. The evolution can be seen in Figure 4. The peak of the thermal conductance for sufficiently high temperatures widens substantially and can even split into two peaks for small couplings (smaller or equal than −0.05 eV). This quantity can also reverse the previous trend and starts to grow slowly for larger temperatures (again, for smaller couplings, now shown in Figure 4).

The Seebeck coefficient and the figure of merit follow similar but not quite the same trends as the previous two quantities. Their evolution is shown in Figure 5, where it can be seen that, as a function of temperature, the maxima and minima of the Seebeck coefficient widen, separate and slightly grow (for smaller couplings, such as −0.05 eV, the height is even kept roughly constant). The maxima of ZT also widen and separate; however, their height clearly increases as a function of temperature. The primary feature responsible for such evolution is the Seebeck coefficient, whose magnitude is enhanced by the square in the expression of ZT, while *G* and κ follow similar trends and due to the Wiedemann–Franz law give a contribution that is roughly proportional to 1/T (which cancels the *T* in the numerator or ZT).

### 3.2. Wedge–Wedge Configuration

This configuration consists of two protuberances or wedges facing each other across the gap. Such a setup gives rise to a transmission that might look similar to that of the previous case, i.e., it has again a series of resonances (one or two in case of spin splitting) generated by localized states. However, these resonances are not the same as those from the previous case and lead to different trends in the thermoelectric coefficients, as we shall see. Note again that we consider here only non-magnetic configurations, which should allow to more clearly distinguish both types of structural configurations and univocally assign to each of them a type of evolution of the thermoelectric coefficients. We will only consider, for this configuration, as with the previous one, a single resonance in the transmission whose shape or position does not depend on any magnetic configuration of the electrodes [31].

The main differences with the previous configuration are due to the height and sharpness of the peak of the transmission, i.e., the resonance is higher and more pronounced (thinner) in this case. This is due to the symmetric nature of this configuration, because here there are localized states at the same energy on both sides of the gap, and the probability of transmission is higher than in that situation where there is only one state on one of the sides. This configuration is then symmetric, although it does not give rise to Breit–Wigner resonances of height equal to one because both states are coupled asymmetrically to each side of the gap.

As a function of the Fermi energy, the thermoelectric coefficients are similar but not quite the same to the previous case, i.e., they have a similar shape (the conductance has a single peak; the Seebeck coefficient has a maximum and a minimum above and below the on-site energy, respectively; the thermal conductance has a single peak; and the figure of merit has two peaks around the on-site energy) but different heights and widths. The thermoelectric coefficients also show a different evolution with the coupling parameter and the temperature, as we will see, which will allow distinguishing this configuration from the previous one.

#### 3.2.1. Evolution with the Coupling Parameter

**Figure 6:**
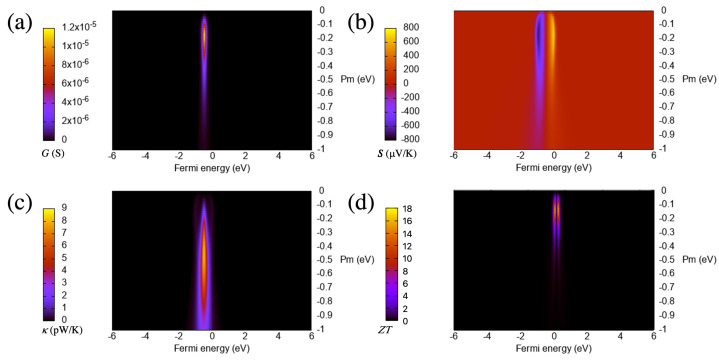
Electrical conductance (**a**), Seebeck coefficient (**b**), electronic thermal conductance (**c**) and figure of merit (**d**) as a function of the Fermi energy and the coupling parameter for the wedge–wedge configuration. The temperature is 300 K.

**Figure 7:**
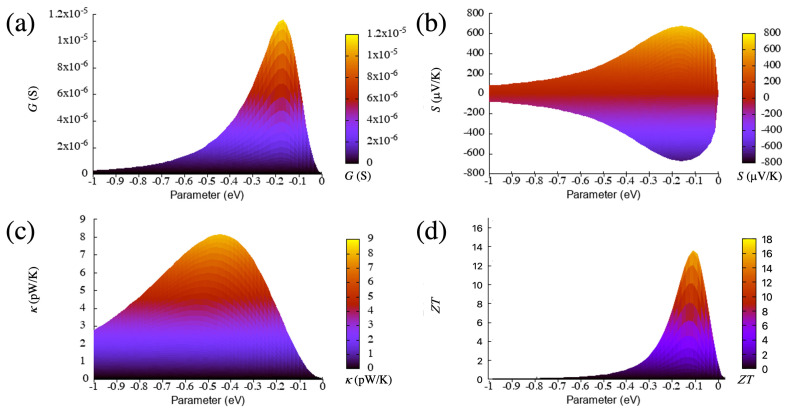
Electrical conductance (**a**), Seebeck coefficient (**b**), electronic thermal conductance (**c**) and figure of merit (**d**) as a function of the coupling parameter for the wedge–wedge configuration (side view of Figure 6). The temperature is 300 K.

The main parameter that influences the thermoelectric properties is again the coupling between the surface states and the bulk states, $t_{l(r),1d\sigma }$ (which, in this case, appears on both sides in this configuration). We assume that the junction is symmetric, i.e., the type of material and structural configuration (size and shape of the wedge or protuberance) are the same on both sides, and therefore the coupling parameters are equal. We then evolve equally the coupling parameters on both sides to study the behavior of the thermoelectric coefficients. The evolution of most of the thermoelectric quantities and, in particular, the Seebeck coefficient and the figure of merit, with this parameter is again not evident, as can be seen in Figure 6 and Figure 7, respectively, where we plot such quantities as a function of this parameter and energy.

Both of these quantities have high values (in absolute terms), which are clearly higher than those in the previous case. Furthermore, the evolution, although similar in certain ranges, is not exactly the same as that of the previous configuration and can be used to distinguish both configurations. We describe in detail the characteristics of such evolution and the differences with the previous configuration in the following.

We focus first again on the electrical conductance, whose evolution can also be captured by taking as reference Equation (9) and deriving the thermoelectric coefficients from it. On the one hand, the calculated conductance in this configuration steadily increases from zero, such as in the wedge–edge case but keeps growing slightly until moderate magnitudes of the coupling (∼−0.1 eV) as can be seen in Figure 7. This means that the maximum moves to larger absolute values of the coupling. The behavior for large magnitudes of the couplings is also different from that of the wedge–edge configuration, where the conductance decreases slowly with it.

In this case, the decrease is more pronounced. On the other hand, the values of this quantity are much higher than those in the previous case, almost two orders of magnitude larger. This shows that, when using a simple transport property, such as the electrical conductance, its evolution with certain parameters and its magnitude, it is possible to distinguish and characterize different nanogap configurations.

The absolute value of the Seebeck coefficient also has two maxima (i.e., it has a maximum and a minimum above and below the on-site energy, respectively), such as in the wedge–edge configuration. This parameter steadily grows as the coupling increases from zero but then slowly decreases as can be seen in Figure 7. The maxima also move to larger absolute values of the coupling, and the values are two-times larger than in the previous configuration. This coefficient provides another example of quantity that can be used to distinguish both types of nanogap configurations and shows again that, although the transmissions are similar (one or various peaks), the quantities derived from it behave differently as a function of certain parameters. This proves as well that those quantities can be sensitive to very small changes derived from different structures or compositions on both sides of the gap.

The thermal conductance has a single peak for most of the range of the coupling parameter (see Figure 6), as opposed to the previous case, which had two peaks for a certain range (small couplings) and a wider structure. The growth and decline of this quantity is more pronounced than in the wedge–edge case but somehow similar as can be seen in Figure 7. The maximum, however, is slightly more pronounced and moves slightly to smaller absolute values of the coupling. This evolution is due again to the different magnitude and shape of the resonance, which produces larger absolute values of this quantity (roughly one order of magnitude larger than in the previous case).

The figure of merit, on the other hand, has a clear maximum as a function of the coupling parameter and initially increases more smoothly than in the wedge–edge configuration as can be seen in Figure 7. This behavior is again due to the dependence of the figure of merit on other variables, such as the electronic and thermal conductances and the Seebeck coefficient. The first two quantities increase first with the coupling; however, the increase is more pronounced for the thermal conductance; this, along with the small decrease of the Seebeck coefficient, gives rise to the maximum of the figure of merit as a function of the coupling parameter, after which, this quantity decreases again sharply (but not as much as in the previous case).

The maximum moves as well to larger absolute values of the coupling, such as in the electrical conductance and the Seebeck coefficient. Note also that, in this case, the magnitude of the figure of merit is much higher than in the wedge–edge configuration, with values larger than 200. This implies that higher thermoelectric efficiencies can be achieved with symmetric configurations that lead to higher resonances in the transmission.

#### 3.2.2. Evolution with Temperature

The evolution with temperature of the thermoelectric coefficients is also shown in Figure 4 and Figure 5. This evolution is similar to that of the wedge–edge configuration; however, the exact dependencies of certain coefficients are different. In the case of the electrical conductance, its evolution is similar and almost indistinguishable to that of the previous configuration. However, the total magnitude of this quantity is much higher (almost two orders of magnitude). The thermal conductance, however, decreases more steadily and has a maximum that does not widen too much as the temperature increases, keeping, in this case, an evolution that looks similar to that of the electrical conductance. This shows that the more symmetric the coupling is, the more similar the evolution of both quantities.

In the case of the Seebeck coefficient, the increase of the height and the separation of the maxima/minima are more pronounced than they are in the previous configuration, and the absolute values are larger as well. This is a consequence of the less asymmetric configuration, produced by the presence of two localized states with the same energies that couple across the gap. This symmetry increases the magnitude of the peak and, therefore, the range where the derivative is high. The same behavior can also be seen for the figure of merit, whose peaks clearly have a more pronounced increase than in the previous configuration and reach higher values. This shows that the more symmetric the configuration is in these systems, the higher the thermoelectric performance.

## 4. Conclusions

We thoroughly characterized the thermoelectric properties of nanoscale junctions based on physical gaps between the surfaces or edges of two-dimensional materials. We found that, depending on the type of feature present on one or both sides of the gap, the thermoelectric coefficients can be different and have distinct evolutions with certain parameters. In particular, we found that all coefficients showed a non-trivial behavior as a function of the coupling parameter between the localized states and the bulk states of the electrodes. The electrical and thermal conductances, the figure of merit and the absolute value of the Seebeck coefficient increased first and then decreased as a function of the absolute value of the coupling parameter.

These increases and decreases depended on the type of configuration: for the wedge–edge configuration, the increases of the electrical conductance, the Seebeck coefficient and the figure of merit were sharper for small couplings, while, for the thermal conductance, this was smoother; the decreases of all these quantities except for the figure of merit were smoother in this case. The position of the maxima also depended on the configuration, i.e., for the wedge–edge case, all these quantities except for the thermal conductance had maxima located at smaller values of the coupling. Regarding the total magnitude of the coefficients, this was much higher in the wedge–wedge case for all of them. Such evolutions can be qualitatively explained and characterized by using a simple model of an asymmetric Breit–Wigner resonance.

We also considered the evolution with temperature of the thermoelectric coefficients. This parameter, which enters into the Fermi distribution function, had a sizeable influence on the absolute magnitude of these quantities. In particular, we found a dissimilar evolution with temperature: increasing the temperature substantially decreased the magnitude of the electrical and thermal conductances but increased the magnitude of the Seebeck coefficient and the figure of merit. We conclude then that the higher the temperature, the better the thermoelectric performance. Regarding the differences between configurations, the increase or decrease of all coefficients with temperature was larger and more pronounced in the wedge–wedge case.

The results also show that it is possible to distinguish structural configurations (wedge–edge or wedge–wedge) of a given gap with the thermoelectric coefficients. Although the evolutions in both cases were qualitatively similar, there were clear quantitative differences in the magnitude and in other values, such as the the maxima of such evolutions. We then conclude that symmetric configurations with localized states on both sides (wedge–wedge) gave much higher thermoelectric performances as shown by the larger values (one order of magnitude) of the figure of merit in such cases.

Finally, we note that the results presented here not only apply to surfaces or edges separated by a gap but also to many other systems, such as those that have nanoscale objects connected between electrodes and that, in principle, can be considered as qualitatively different from those studied here. In those cases, there might appear similar features in the transmission (small and sharp resonances) that come, for instance, from surface states or states localized near the contacts. Such features also give rise to similar transport properties (see, e.g., [45]) and lead to qualitatively similar thermoelectric results.

## Figures and Tables

**Figure A1:**
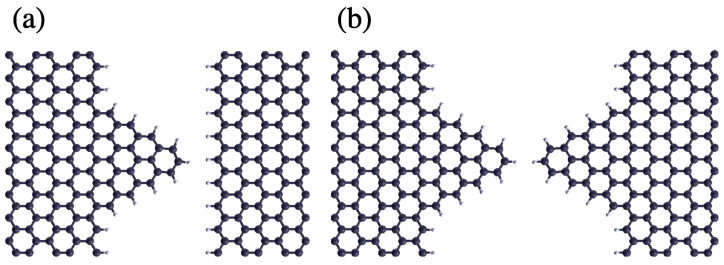
Atomic coordinates used to run the ab initio simulation in the wedge–edge (**a**) and wedge–wedge (**b**) configurations. Black and white spheres represent carbon and hydrogen atoms, respectively.

**Figure A2:**
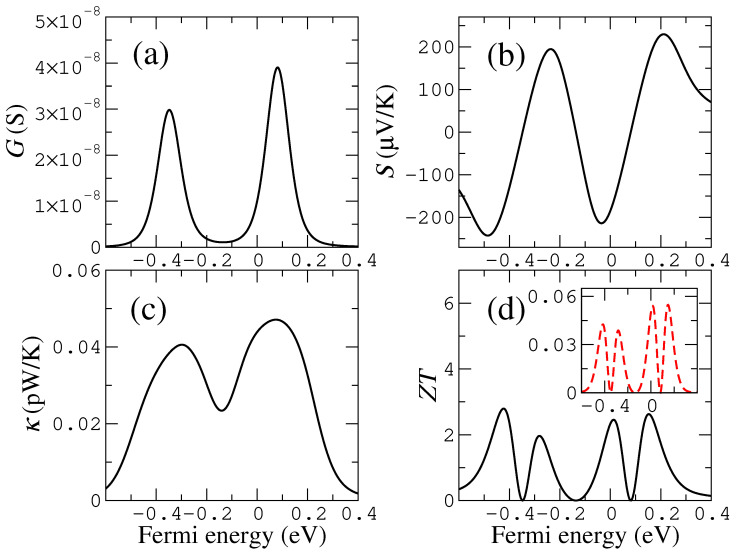
Electrical conductance (**a**), Seebeck coefficient (**b**), thermal conductance (**c**) and figure of merit (**d**) for a graphene wedge facing a straight edge. The inset shows the figure of merit calculated with a phonon thermal conductance of 2 pW/K.

**Figure A3:**
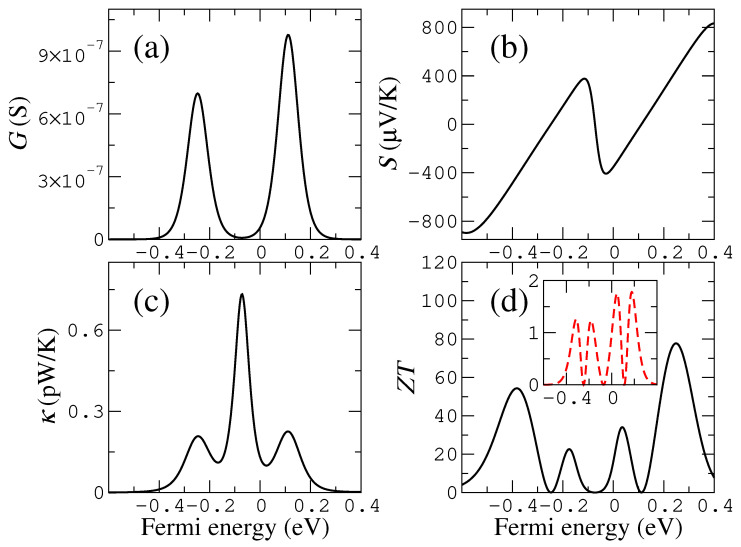
Electrical conductance (**a**), Seebeck coefficient (**b**), thermal conductance (**c**) and figure of merit (**d**) for a graphene wedge facing another wedge. The inset shows the figure of merit calculated with a phonon thermal conductance of 2 pW/K.

## Data Availability

The data supporting the reported results can be provided by email.

## Appendix A. Nanogaps in Graphene Layers: Ab-Initio Results

In order to compare with the results of the model, we also simulated the thermoelectric properties of graphene layers separated by a gap. We used density functional theory [46], as implemented in the Siesta code [47], which employs norm-conserving pseudopotentials and linear combinations of atomic orbitals. We used a double-zeta (DZ) basis set, the local density approximation (LDA) [48] and a real space grid defined with an energy cut-off of 300 Ry. We relaxed the coordinates of the carbon and hydrogen atoms at the edges until the forces were smaller than 0.05 eV/Å.

The number of atoms, shown in Figure A1, were 226 and 254 for the wedge–edge and wedge–wedge configurations, respectively. The size of the gap at the thinnest point, defined as the distance between the terminating hydrogen atoms at the edges or at the tip of the wedge, was 2 Å. Once the Siesta calculation finished, the Hamiltonian and overlap matrices were used to calculate the thermoelectric properties with the Gollum [45] code with a temperature of 300 K.

The phonon thermal conductance was approximated by taking a value of the thermal conductance per unit area of about $10^{8}$ W/(Km${}^{2}$) [39] and an area of 0.02 nm${}^{2}$ that encompasses the hydrogen atom at the tip of the wedge, which gave $\kappa_{\mathrm{ph}}=2$ pW/K. Notice also that, in this case, since the graphene edges were magnetic, the resonances were split in two. This implies that both spin components, which can have different transmissions $T^{↑}\left(E\right)$ and $T^{↓}\left(E\right)$, have to be used in the thermoelectric expressions:

(A1) $$G=\frac{e^{2}}{h}L_{0}^{t}$$

(A2) $$S=-\frac{1}{eT}\frac{L_{1}^{t}}{L_{0}^{t}}$$

(A3) $$\kappa =\frac{1}{hT}\left(L_{2}^{t}-\frac{L_{1}^{t2}}{L_{0}^{t}}\right)$$

(A4) $$ZT=\frac{1}{\frac{L_{0}^{t}L_{2}^{t}}{L_{1}^{t2}}-1}$$

where $L_{i}^{t}=L_{i}^{↑}+L_{i}^{↓}$. The use of both spin components in such expressions gives thermoelectric coefficients that are roughly the sum of the contribution of each resonance if they are away from each other as can be seen in Figure A2 and Figure A3. In certain cases, the resulting coefficients mix the contribution of both resonances and lead to dependencies that are not clearly split such as the thermal conductance in Figure A3. In any case, the results for each resonance qualitatively agree with those predicted by the model. Notice as well that, even though these particular systems based on graphene layers were not particularly tailored to produce large thermoelectric coefficients, the resulting thermoelectric performances were still good, particularly those of the wedge–wedge case, which again shows the potential of these systems as future elements in thermoelectric devices.
